# Association between Clinical Manifestations in Temporomandibular Joint Disorders and Corresponding Radiographic Findings

**DOI:** 10.3390/jcm13164886

**Published:** 2024-08-19

**Authors:** Barbara Wojciechowska, Arkadiusz Szarmach, Adam Michcik, Tomasz Wach, Barbara Drogoszewska

**Affiliations:** 1 Department of Maxillofacial Surgery, Medical University of Gdansk, 17 Mariana Smoluchowskiego Street, 80-214 Gdansk, Poland; adammichcik@gumed.edu.pl (A.M.); drog@gumed.edu.pl (B.D.); 2 2nd Department of Radiology, Medical University of Gdansk, 17 Mariana Smoluchowskiego Street, 80-214 Gdansk, Poland; arkadiusz.szarmach@gumed.edu.pl; 3 Department of Maxillofacial Surgery, Medical University of Lodz, 113 Żeromskiego Street, 90-549 Lodz, Poland

**Keywords:** temporomandibular joint disorders, ultrasonography, cone-beam computed tomography, magnetic resonance imaging

## Abstract

**Background**: Temporomandibular disorders are common conditions characterized by discomfort within the temporomandibular joints, acoustic changes, and restricted mandibular movement. Accurate diagnosis and subsequent treatment rely heavily on clinical examination, but it is often necessary to add radiological examinations to the diagnostics. Magnetic Resonance Imaging (MRI) is the gold standard for visualizing the disc, while cone-beam computed tomography (CBCT) is primarily used for evaluating condylar morphology. Ultrasound (US) serves as a real-time imaging modality for soft tissues. The objective of the present study was to explore the association between clinical manifestations observed in patients with temporomandibular joint disorders and corresponding radiographic findings. **Methods**: A total of 63 adult patients (51 female and 12 male) with temporomandibular joint disorders were included in this cross-sectional study. Each patient underwent a clinical examination, followed by appropriate radiological examinations (MRI, CBCT, or US). The level of statistical significance was set at an alpha of 0.05. The Shapiro–Wilk test assessed the normality of numerical variables. The Wilcoxon rank sum test compared two independent groups with non-normally distributed data. Relationships between categorical variables were evaluated using the Pearson chi-square test or Fisher’s exact test. The Kendall tau (τ) method analyzed the correlation between two binary variables. **Results**: The analysis included 63 patients with TMD, predominantly females (80.95%). Ages ranged from 18 to 74 years with a median of 39 years. In the CBCT study, we observed rarefied changes in the left bone structures in patients with bruxism (*p* = 0.010). MRI and ultrasound imaging revealed changes in patients with limited jaw opening: erosions in the right mandibular head on ultrasound (*p* = 0.008) and abnormal right bone structures on MRI (*p* = 0.009). In CBCT, asymmetry in the left joint space was correlated with a high incidence of right side muscle tension (*p* = 0.004). Additionally, both CBCT and ultrasound showed a correlation between muscle tension and erosion (*p* = 0.040 in ultrasound, *p* = 0.020 in CBCT). Acoustic changes, when compared with radiological imaging, were evident in all three studies, like temporomandibular joint pain or palpation. **Conclusions**: Our study compared three radiographic imaging methods with clinical examinations to assess their correlation with clinical symptoms. Each imaging technique provided unique insights depending on the specific symptoms presented. The observed correlations varied, highlighting the unique contributions of each modality to the diagnostic process. This underscores the importance of employing multiple diagnostic approaches for a thorough assessment of the temporomandibular joint. However, a limitation of our study is the small sample size and the uneven distribution of participants among the groups. Additionally, not all patients underwent every imaging modality.

## 1. Introduction

Temporomandibular disorders (TMDs) are defined as a group of musculoskeletal and neuromuscular conditions involving the temporomandibular joint (TMJ), the masticatory muscles, and the associated structures [1,2]. TMDs affect as many as 34% of the population, highlighting a significant public health issue. TMDs have a higher prevalence in women compared to men. The prevalence of TMD varies by age, revealing its widespread impact: 27% among individuals up to 18 years old, 41% among those aged 18–60 years, and 36% among those over 60 years of age [2,3,4,5]. The etiology is multifactorial and includes biological, environmental, social, emotional, and cognitive triggers. Biological aspects encompass genetic predispositions, hormonal influences, and anatomical variations [2,6,7,8]. Environmental factors include parafunctional habits such as bruxism and exposure to physical stressors. Bruxism is characterized by the repetitive contraction of the masticatory muscles. It results in tooth clenching or grinding, associated with bracing or thrusting (projecting forward or sideways) of the mandible. The literature describes a strong association between bruxism, both awake and sleep bruxism, and the development of temporomandibular disorders (TMDs). Studies suggest that individuals with bruxism are at a higher risk of developing TMD in the future [9,10]. Social determinants involve interpersonal relationships and lifestyle choices, which modulate stress levels. Emotional factors, including anxiety, depression, and psychological stress, play a pivotal role in the onset and exacerbation of TMD symptoms. Cognitive aspects, such as coping mechanisms and pain perception, significantly influence the development and progression of the disorder [2,6,7,8]. Excessive joint loading results in increased production of extracellular matrix. The remodeling process may be modulated by concomitant systemic pathologies, including autoimmune diseases, endocrine disorders, nutritional deficiencies, metabolic syndromes, infectious diseases, pharmacological treatments, and advanced age, which diminishes the adaptive capacity of the joints. The most common joint pathology affecting the TMJ is degenerative joint disease (DJD), also known as osteoarthrosis (TMJ OA) or osteoarthritis [11]. TMJ OA is characterized by chondrocyte death, extracellular matrix (ECM) degradation, and subchondral bone remodeling [12]. With advancing age, degenerative changes occur, with DJD being the primary disorder affecting the joint in individuals over 60 years old, impacting up to 70% of this population [11,13].

The most reported symptom by patients is pain localized in the preauricular region. Additionally, acoustic changes, variations in the amplitude of mouth opening, and lateral movements may occur. In more advanced cases, symptoms extend to include headaches, otalgia, and facial pain. Stressful situations can exacerbate the symptoms of the disorder. Some patients remain asymptomatic and pathologies are detected during imaging studies [14,15,16,17].

Clinical examination is fundamental in the diagnostic assessment of temporomandibular joint disorders, providing critical information regarding the functional and structural integrity of the joint [16]. The most used diagnostic protocol in TMD studies is the Research Diagnostic Criteria for Temporomandibular Disorders (DC/TMD) [18]. TMD includes both structural and biopsychosocial elements and the DC/TMD framework evaluates these through two axes. Axis I outlines a protocol for a standardized physical examination to determine specific physical diagnoses of TMD related to the joint and muscles. Axis II encompasses various tools to evaluate the patient’s psychological condition. There are 12 most common diagnoses of TMD described in Axis I of the DC/TMD, divided into pain-related disorders (myalgia, local myalgia, myofascial pain, myofascial pain with referral, arthralgia, headache attributed to TMD) and intra-articular temporomandibular disorders (disc displacement with reduction, disc displacement with reduction with intermittent locking, disc displacement with reduction with limited opening, disc displacement with reduction without limited opening, degenerative joint disease, subluxation) [16,18].

In addition to clinical evaluation, various complementary diagnostic modalities are employed to provide a comprehensive assessment of TMD [16]. Computed tomography (CT) offers detailed cross-sectional imaging of the bone structures, facilitating the identification of osseous abnormalities and joint deformities [19]. Magnetic resonance imaging (MRI) provides superior soft tissue contrast, enabling the evaluation of the articular disc, ligaments, and surrounding soft tissues [16]. Cone-beam computed tomography (CBCT) is particularly useful for its three-dimensional imaging capabilities, allowing for precise anatomical localization and assessment of the joint morphology [16,20]. Conventional panoramic radiography offers a broad view of the TMJ and surrounding structures [21]. Additionally, ultrasonography (US) provides real-time imaging of soft tissue structures [16]. Together, these diagnostic tools enhance the accuracy of diagnosis, guide treatment planning, and monitor the progression of temporomandibular joint disorders [16,21]. Changes observed in the TMD include alterations in shape and size of joint components, specifically, flattened fossa, less pronounced articular eminence, decreased condylar volume, and thickened disc [11,13].

There is a lack of reports in the literature comparing clinical symptoms with radiological findings using all three imaging methods. Additionally, comprehensive analyses that compare clinical symptoms with radiographic findings across a varied patient population are deficient. Our study provides a comprehensive perspective by examining how different imaging techniques relate to specific clinical symptoms. By evaluating and comparing these three radiological methods, we aim to determine their effectiveness in capturing details relevant to various symptoms. We hypothesize that there is a significant association between the clinical manifestations of temporomandibular joint disorders and corresponding radiographic findings using CBCT, MRI, and US. Furthermore, different imaging techniques may provide varying levels of detail relevant to specific clinical symptoms.

The aim of the study was to explore this association and determine which radiological examinations offer the most information for specific clinical symptoms.

## 2. Materials and Methods

A total of 63 patients with temporomandibular joint disorders were enrolled in this study. Subjects gave consent to participate in the study. The study was approved by the Ethics Committee of the Medical University of Gdańsk, Poland.

This cross-sectional study was conducted at the Department of Maxillofacial Surgery at the Medical University of Gdańsk, Poland.

The clinical examinations were conducted and considered the inclusion criteria of joint disorders [18]. The inclusion criteria were as follows:

- Patients over 18 years of age;
- Patients who agreed to participate in the study;
- General good health;
- Clinically confirmed diagnosis of temporomandibular joint disorders according to specified diagnostic criteria.
- The exclusion criteria were as follows:
- Patients with a history of TMD treatment (conservative or surgical treatment);
- Patients with polyarthritis (such as rheumatoid arthritis, gout arthritis, and psoriatic arthritis);
- Patients with cervical and neuropathic pain;
- Patients with a history of orthodontic treatment, plastic surgery, or other craniofacial surgery;
- Patients with a history of trauma to the head region.

Each patient included in the study underwent a comprehensive clinical examination conducted by one person.

The clinical examination included the following:

Medical history: duration of symptoms, comorbidities, pain symptoms, tinnitus, headaches, tooth pain, bruxism, clenching.

Physical examination: assessment of dental status, occlusion planes, orthodontic abnormalities; evaluation of the temporomandibular joints for clicks, crepitus, and deviations; palpation and spontaneous pain of the joints; jaw opening path and range; evaluation of the masticatory muscles for tenderness, tension, and possible hypertrophy.

Depending on the reported symptoms, after clinical examination, patients also received appropriate radiological assessments including ultrasonography, cone-beam computed tomography, and magnetic resonance imaging. The radiological examinations were described by one radiologist who was unaware of the clinical examination results. Additionally, panoramic radiography was evaluated to assess dental status and potential changes in the surrounding tissues.

**Figure 1:**
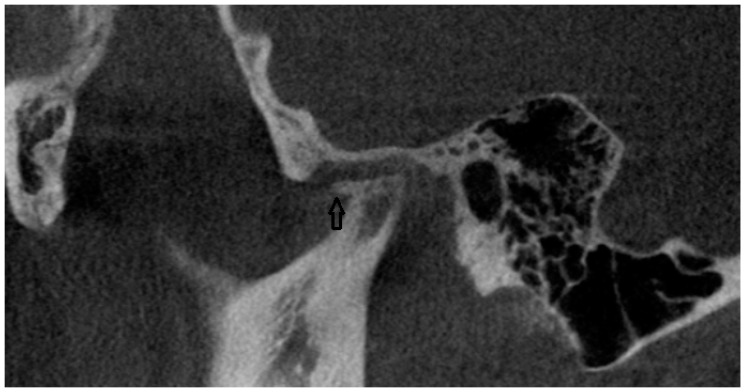
CBCT scan (black arrow points to osteophyte).

In the CBCT analysis, a comprehensive assessment was conducted on the morphology of the condylar process. This evaluation adhered to established diagnostic criteria aimed at identifying various pathological changes. Specifically, the analysis included the detection and characterization of osteophytes or erosions, indicating localized bone loss or damage, and the presence of cystic formations was evaluated (Figure 1). The study involved a detailed examination of the bone structure itself. This included assessing regions for possible rarefaction or densification. The evaluation extended to the measurement of the joint space width. The condylar fossa and the articular tubercle were also assessed. All CBCT examinations were performed in layers 0.2 mm thick, with 90 kV and 40 mAs using a Carestream CS 9300 (Kodak Dental Systems, San Francisco, CA, USA).

**Figure 2:**
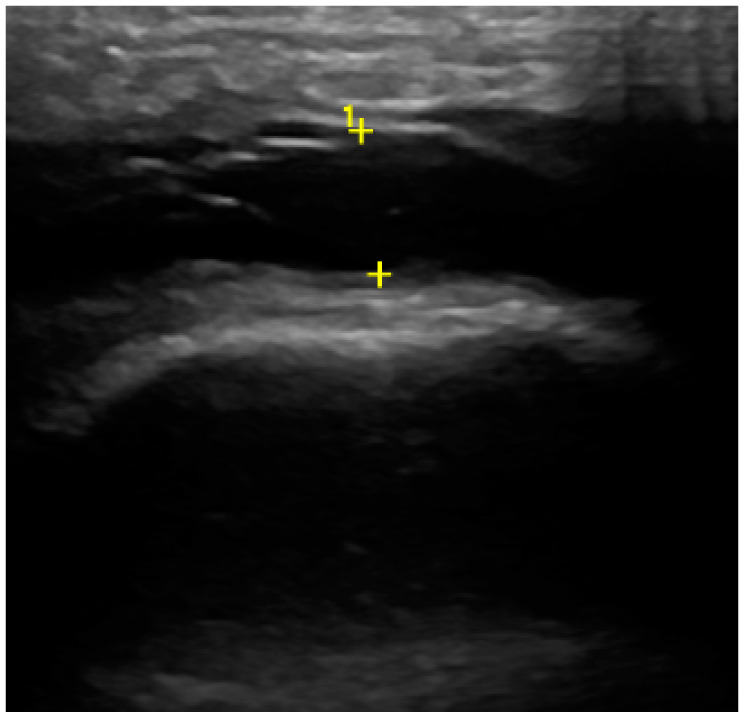
Ultrasound (effusion within the joint).

**Figure 3:**
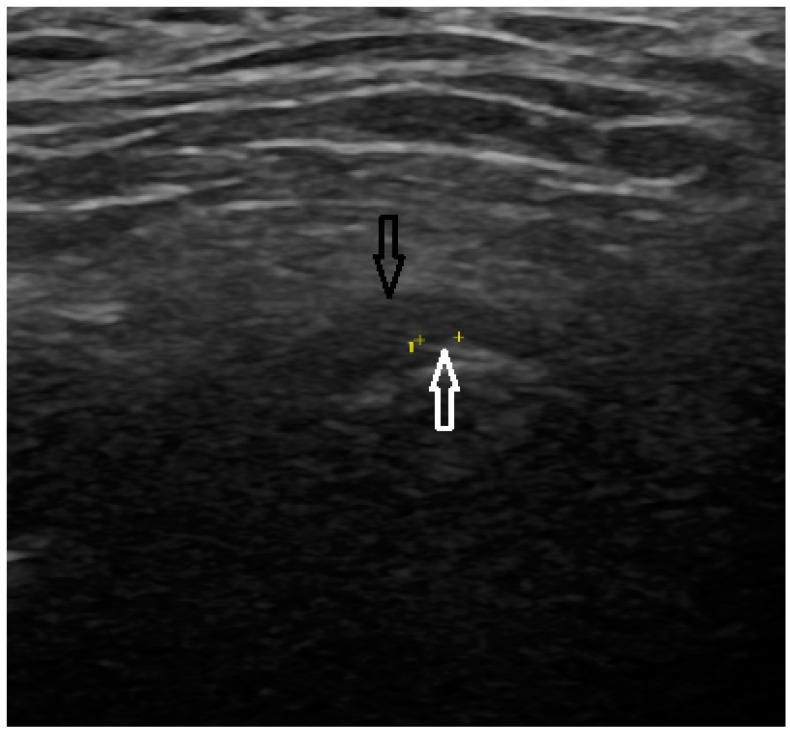
Ultrasound (articular disc—black arrow, osteophyte—white arrow).

In the US analysis, we aimed to assess synovial effusion within the joint, which is indicative of inflammation or pathological fluid accumulation (Figure 2). Additionally, a thorough examination of the osseous structures was conducted. This included identifying the presence of osteophytes and evaluating any erosive changes to the bone surfaces. Furthermore, the analysis extended to the evaluation of the articular disc (Figure 3). For the purposes of the study, a unique research protocol was proposed, consisting of the assessment of the temporomandibular joint in the neutral position (mouth closed) with the mouth in maximum opening and the assessment of the path of the articular disc movement in a dynamic test. All examinations were performed by the same experienced radiologist using a LOGIQ E10 ultrasound with a dedicated L8-18i hockey stick linear probe (GE Healthcare, Boston, MA, USA).

**Figure 4:**
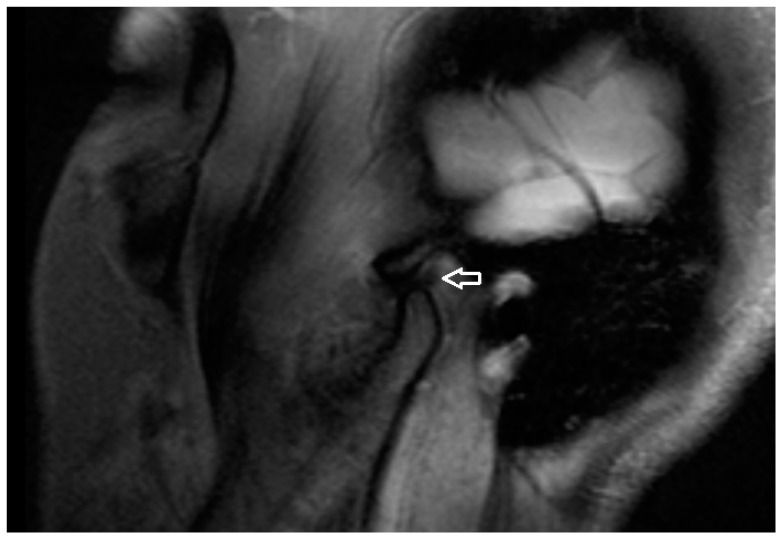
PD TSE SPAIR Sagittal MRI scan (articular disc—white arrow).

All MRI examinations were performed using a 3T MRI scanner (MAGNETOM Vida, Siemens AG, Erlangen, Germany) with a dedicated head coil. A special protocol (Table 1 consisting of three scanning phases was prepared for the study: 1. mouth closed, 2. mouth opening 2 cm (a dilator was used), 3. mouth maximally opened. In the MRI examination, a detailed analysis was performed on the TM joint structures. The presence of osteophytes and erosive changes to the bone surfaces were also assessed, as these can indicate progressive joint damage and contribute to the severity of the condition. Additionally, the condition of the articular disc was examined for any signs of displacement or degeneration, while the presence of synovial effusion was evaluated to determine the extent of joint inflammation and fluid accumulation (Figure 4).

In the CBCT examination, the bony elements of the TMJ as well as the adjacent soft tissues have been evaluated. CT is perfect for the evaluation of fractures, degenerative changes, erosions, infection, invasion by tumor, as well as congenital anomalies.

During the MRI examination, the soft tissue structures of the TMJ (articular disc, synovial membrane, lateral pterygoid muscle) disc displacements were assessed. MRI could also detect the early signs of TMJ dysfunction, like thickening of the anterior or posterior band, rupture of retrodiscal tissue, changes in shape of the disc, or joint effusion.

Ultrasound enabled us to look for the presence of a joint effusion. It is also used to evaluate cartilage as well as disk displacement with both open and closed mouth imaging.

All examinations were performed using the same equipment (CBCT, MRI, and USG) by the same radiologist with over 10 years of experience in masticatory imaging diagnostics. At the same time, the radiologist was not informed about the results of the clinical examination or the initial diagnosis.

The level of statistical significance was established at an alpha of 0.05. The normality of numerical variables was assessed using the Shapiro–Wilk test. Variables that deviated from a normal distribution were summarized using the median (Mdn) and the interquartile range, specifically the first (Q1) and third (Q3) quartiles. Counts (*n*) and proportions (%) of each category were provided for categorical variables.

The Wilcoxon rank sum test was utilized when comparing two independent groups with non-normally distributed numerical data. The relationship between two categorical variables was evaluated using the Pearson chi-square test or Fisher’s exact test depending on the expected frequencies within the contingency tables.

In the analysis of the contingency table, with a singular degree of freedom, the phi coefficient (ϕ) was employed as the measure of effect size.

A power analysis was performed to ascertain the sufficiency of the sample size required to detect a predefined effect size utilizing a chi-squared distribution with one degree of freedom. The objective was to achieve a statistical power of 0.8 (1−β) and a significance level of 0.05 (α) employing Cohen’s w of 0.40, which is equivalent to ϕ in a 2 × 2 contingency table. The analysis indicated that a sample size of approximately 50 observations is necessary to satisfy these parameters.

For analyzing the correlation between two binary variables, we employed the Kendall tau (τ) method. This rank-based measure of association was quantified and the significance of the correlation was determined using the *p*-value from the asymptotic approximation of the Wald z test statistic.

Analyses were conducted using the R Statistical language (version 4.3.1; R Core Team, 2023) on Windows 10 pro 64 bit (build 19045).

## 3. Results

The analysis encompassed the results of 63 adult patients presenting with TMD. Regarding gender distribution, the sample was predominantly females, comprising 80.95% (51/63) of the total, compared to 19.05% (12/63) males (Table 2). The patients’ ages ranged from 18 to 74 years with a median age of 39 years, a first quartile (Q1) at 28.50 years, and a third quartile (Q3) at 54.50 years.

We focused on describing the correlations between teeth grinding, limited jaw opening, masticatory muscle tension, acoustic changes, pain, and palpation discomfort with radiological findings.

### 3.1. Teeth Grinding

In CBCT, the data indicate that when the left bone structures are normal, 40.00% (*n* = 2) of these cases are associated with teeth grinding, compared to a higher prevalence of 88.37% (*n* = 38) where no teeth grinding is reported (*p* = 0.027, ϕ = 0.33). In rarefied left bone structures, 60.00% (*n* = 3) of the cases exhibit teeth grinding, compared to just 6.98% (*n* = 3) of cases without teeth grinding (*p* = 0.010, ϕ = 0.41).

### 3.2. Limited Mandibular Opening

In the US, the data indicated that 33.33% (n = 4) of patients with erosions in the right mandibular head experienced limited mandibular opening compared to only 2.50% (*n* = 1) who did not (*p* = 0.008), suggesting a moderate to strong positive association (ϕ = 0.44) (Table 3).

In the case of MRI, limited mandibular opening occurred in 11.11% (*n* = 1) of patients with normal right bone structures against a backdrop of 62.16% (*n* = 23) who did not exhibit limited mandibular opening (*p* = 0.009), with a ϕ = 0.41 indicating a moderate association. Limited mandibular opening was observed in 88.89% (*n* = 8) of patients with abnormal right bone structures compared to 37.84% (*n* = 14) of those without such abnormalities (*p* = 0.009, ϕ = 0.41). A total of 33.33% (*n* = 3) of patients with right disc dislocation exhibited limited mandibular opening, whereas only 5.41% (*n* = 2) of patients without disc dislocation showed this symptom (*p* = 0.044, ϕ = 0.36).

### 3.3. Masticatory Muscle Tension

In CBCT, patients with normal left joint space showed a lower prevalence of right side masticatory muscle tension (22.22%, *n* = 2) compared to those without muscle tension (66.67%, *n* = 26) (*p* = 0.024, ϕ = 0.35) (Table 4). A total of 66.67% (*n* = 6) of patients with narrowed left joint space experienced right side muscle tension compared to only 23.08% (*n* = 9) who did not (*p* = 0.018, ϕ = 0.37). Asymmetry in the left joint space resulted in a high incidence of right side muscle tension (66.67%, *n* = 6) compared to those without tension (15.38%, *n* = 6) (*p* = 0.004) with moderate to strong association, ϕ = 0.46. The presence of erosions in the right mandibular head was associated with right side masticatory muscle tension in 55.56% (*n* = 5) of cases versus 15.38% (*n* = 6) without tension (*p* = 0.020, ϕ = 0.37). Normal left bone structures were associated with a lower incidence of right side muscle tension (55.56%, *n* = 5) compared to a high prevalence among those without muscle tension (89.74%, *n* = 35) (*p* = 0.031, ϕ = 0.36).

Within US observations, erosions in the right bone structures were associated with a higher rate of right side muscle tension (37.50%, *n* = 3) compared to a lower rate (6.82%, *n* = 3) among those without such tension (*p* = 0.040, ϕ = 0.35).

Asymmetry in the left joint space in CBCT was associated with left side muscle tension observed in 58.33% (*n* = 2) of patients compared to 13.89% (*n* = 3) without such tension (*p* = 0.042, ϕ = 0.29).

### 3.4. Acoustic Changes

A total of 34.78% (*n* = 8) of patients with erosions on the left mandibular head as detected by CBCT exhibited erosions, compared to none in the group without acoustic symptoms (*p* = 0.001, ϕ = 0.47) (Table 5).

In ultrasonography, a normal outline is associated with a higher incidence of acoustic changes (83.33%, *n* = 20) on the outline of the right mandibular head compared to those with an abnormal outline (16.67%, *n* = 4) (*p* = 0.041, ϕ = 0.28).

Patients with degenerative changes in the right joint exhibit acoustic symptoms at a rate of 33.33% (*n* = 8), compared to 60.71% (*n* = 17) who do not (*p* = 0.049, ϕ = 0.27).

A homogeneous right articular disc is associated with a higher prevalence of acoustic changes on the left (65.22%, *n* = 15) compared to when the disc is heterogeneous (34.48%, *n* = 10) (*p* = 0.028, ϕ = 0.31) (Table 6). A homogeneous disc is correlated with acoustic changes on the left with an occurrence rate of 90.00% (*n* = 18) compared to 10.00% (*n* = 12) when the disc is heterogeneous (*p* = 0.002, ϕ = 0.46).

There are erosions in 35% (*n* = 7) of patients with right joint clicks, compared to 3.57% (*n* = 1) in those without clicks (*p* = 0.006, ϕ = 0.42).

It can be observed that normal left bone structures as assessed by US are present in 90% (*n* = 18) of patients with clicks in the right joint, compared to 65.63% (*n* = 21) in those without clicks (*p* = 0.048, ϕ = 0.28). In the group of patients with left disc displacement, 25% (*n* = 5) exhibited right joint clicks versus only 3.13% (*n* = 1) when not displaced (*p* = 0.026, ϕ = 0.32). None of the patients with a normal left joint space exhibited crepitus in the right TMJ.

Three out of four patients with erosions in the left mandibular head experienced right joint crepitus, resulting in a very high prevalence rate of 75% (*n* = 3) compared to only 11.36% (*n* = 5) among those without erosions in CBCT (*p* = 0.012, ϕ = 0.38).

### 3.5. Pain

The occurrence of pain in the presence of radiologically normal right bone structures is reported in 53.85% (*n* = 14) of patients, while 80.77% (*n* = 21) of those with normal structures do not experience pain (*p* = 0.039, ϕ = 0.30). The presence of erosions in the right bone structures has been associated with a significantly lower incidence of reported pain (23.08%, *n* = 6) compared to those without erosions (*p* = 0.023, ϕ = 0.34). A normal outline of the left mandibular head correlates with a higher prevalence of pain in the right joint (76.92%, *n* = 20) compared to those with abnormalities (46.15%, *n* = 12 do not have pain) (*p* = 0.023, ϕ = 0.34).

Asymmetry in the right joint space in CBCT is associated with pain in the left joint in 40% (*n* = 8) of cases, significantly higher than the 14.29% (*n* = 4) who do not experience pain (*p* = 0.043, ϕ = 0.29) (Table 7). Normal left joint space shows a lower frequency of pain (40%, *n* = 8) compared to a higher percentage (71.43%, *n* = 20) not experiencing pain (*p* = 0.029, ϕ = 0.31). Narrowed left joint space is linked with a higher incidence of pain (50%, *n* = 10) compared to those without pain (17.86%, *n* = 5) (*p* = 0.018, ϕ = 0.34). Asymmetrical left joint space shows a similar pattern to the right joint, where asymmetry is associated with a higher prevalence of pain (40%, *n* = 8 vs. 14.29%, *n* = 4) (*p* = 0.043, ϕ = 0.29).

According to US findings, normal (*n* = 14) and abnormal (*n* = 13) right mandibular head outlines both show significant associations with pain occurrence in the left joint (*p* = 0.002, ϕ = 0.42).

On MRI, heterogeneous discs are more often associated with pain (70%, *n* = 14 vs. 30%, *n* = 6) (*p* = 0.017, ϕ = 0.35).

The presence of erosions in the right mandibular head as detected by CBCT is associated with an increase in palpation discomfort, with 41.18% (*n* = 7) of those with erosions experiencing discomfort compared to only 12.90% (*n* = 4) of those without (*p* = 0.036, ϕ = 0.32) (Table 8).

A normal outline of the left mandibular head as revealed by US is associated with a higher incidence of palpation discomfort in the right side (80.00%, *n* = 16 in cases with a normal outline versus 50.00%, *n* = 16 in those without) (*p* = 0.031, ϕ = 0.30).

MRI findings of erosions in the right bone structures demonstrate a significant association with palpation discomfort, where all individuals with these erosions experienced discomfort (20.00%, *n* = 3) versus none in the absence of erosions (*p* = 0.030, ϕ = 0.38). The presence of disc dislocation on the right side, as determined by MRI, shows a significant correlation with increased palpation discomfort, with 26.67% (*n* = 4) of patients with dislocation reporting discomfort compared to only 3.23% (*n* = 1) without dislocation (*p* = 0.033, ϕ = 0.35).

Asymmetry in the right joint space, as detected by CBCT, correlates with palpation discomfort on the left side, with 50% (*n* = 7) of those with asymmetry experiencing discomfort compared to only 14.71% (*n* = 5) of those without it (*p* = 0.024, ϕ = 0.37) (Table 9). An asymmetrical left joint space noted on CBCT is associated with a corresponding increase in left side discomfort (50%, *n* = 7 with discomfort and 14.71%, *n* = 5 without) (*p* = 0.024, ϕ = 0.37). A normal left joint space seems protective against discomfort, with only 35.71% (*n* = 5) experiencing discomfort compared to 67.65% (*n* = 23) who does not experience it (*p* = 0.041, ϕ = 0.29).

The ultrasound examination of the right bone structures showed that 79.41% (*n* = 27) of patients with normal results reported no discomfort on the left side (*p* = 0.011, ϕ = 0.36). Heterogeneous left articular disc was strongly associated with an increased incidence of discomfort during palpation on the left side, with 77.78% (*n* = 14) of patients experiencing this discomfort (*p* = 0.020, ϕ = 0.32). The presence of erosions in the right mandibular head and right disc edema were linked to a lower incidence of discomfort on the left side, occurring in four cases (22.22%) (*p* = 0.043, ϕ = 0.31). MRI findings showed that 25% (*n* = 3) of patients with erosions in the left bone structures experienced discomfort, whereas none without erosions reported any discomfort (*p* = 0.014), with moderate to strong association ϕ = 0.45.

The prevalence of clinical manifestations was analyzed with stratification by sex. The findings indicated no significant differences in the frequency of these manifestations between males and females except for the “abduction path deviates to the left.” This symptom was significantly more prevalent in males, observed in 33.3% (*n* = 4) of the male cohort compared to 5.88% (*n* = 3) in the female cohort, yielding a *p* = 0.021, ϕ = 0.32.

## 4. Discussion

The primary objective of this study was to examine how clinical symptoms in patients with temporomandibular joint disorders relate to their radiographic findings. By examining the symptoms reported by patients alongside their radiographic images, our research aims to offer a deeper understanding of TMD. This will enhance the ability to predict radiographic abnormalities from clinical signs. In the CBCT study, we observed statistically significant changes in patients with bruxism. MRI and US imaging revealed changes in patients with limited jaw opening. Muscle tension manifested in both US and CBCT. Acoustic changes, when compared with radiological imaging, were evident in all three studies, like temporomandibular joint pain or palpation.

TMD is a commonly occurring disorder affecting up to 34% of the population and presenting symptoms across various age groups [5]. In our study, women constituted a significant majority of the patient cohort, representing 80.95% of the total. This gender distribution aligns with existing literature, which consistently indicates a higher prevalence of TMD among females. The reasons for this gender disparity are not entirely understood, but hormonal differences, particularly the role of estrogen, may play a significant part in the development and exacerbation of the disorder. Research suggests that estrogen might influence TMD in two different ways; high or variable levels may encourage the development of specific forms of TMD, while low levels could intensify other forms. 17-β estradiol (E2) is a factor in the pathogenesis of masticatory organ lesions in women of childbearing age, primarily by inducing MMP9 and MMP13 production in TMJ fibrocartilage, which results in its degradation. However, the specific mechanisms underlying E2’s destructive effects remain poorly understood. Estrogens modulate mRNA replication and Nav1.7 protein expression in the sodium channels of the trigeminal nerve ganglion, thereby lowering the nociceptive threshold of the TMJ and enhancing pain response. The increased number of estrogen receptors in the TMJ of female patients may lead to joint laxity, with receptor polymorphisms potentially influencing 7,l outcomes [8,22,23]. During pregnancy and throughout the menstrual cycle, hormonal fluctuations can lead to increased pain in patients with TMD. Reduced estrogen levels following menopause contribute to TMJ lesions by impairing the synthesis of proteoglycans, collagen, and proteins in the articular cartilage and mandibular disc. This reduction may also exacerbate DJD, osteoporosis, and lead to alveolar bone loss [2,3,4,8,22,23,24].

The key elements in diagnosing temporomandibular joint disorders are a comprehensive clinical evaluation and an extensive clinical history [16]. This should include an assessment of parafunctional activities, dysfunctions, comorbid conditions (particularly depression and anxiety), and lifestyle stressors. The considered chronic orofacial pain lasting for more than 6 months can contribute to a decline in quality of life, social withdrawal, and depressive disorders, burdening the healthcare system. The chronicity of TMD is evident in our sample, with 63.49% of patients experiencing symptoms for over six months. This high prevalence of long-term cases underscores the disorder’s persistent nature and suggests potential delays in seeking effective treatment or challenges in managing the condition. Chronic TMD can lead to progressive worsening and significant impact on patients’ quality of life [18,25].

Panoramic radiography is frequently employed as an initial imaging technique to evaluate dentition and associated structures due to its cost-effectiveness, minimal radiation exposure, and clear visualization of the joint eminence. However, its low sensitivity and specificity compounded by image overlap and the inherent limitations of two-dimensional imaging render it less suitable for diagnosing temporomandibular joint disorders [26]. Schroder et al. [27] noted that panoramic radiography could still be used for assessing potential degenerative changes in the joints. Given its limited diagnostic value, panoramic radiography was not used for TMD diagnostics, reserving it instead for evaluating dentition and bony changes as well as excluding potential odontogenic pathologies such as cysts or tumors [21,26]. Fang et al. [28] evaluated degenerative changes using cephalometric radiographs, which are commonly used in orthodontics. Due to the exclusion of patients undergoing or planning orthodontic treatment, TMJ conditions were not assessed via cephalometric imaging [21,28].

When degenerative changes are suspected, cone-beam computed tomography is the preferred imaging modality. CBCT is employed to assess the bony cortices of the condylar head and the glenoid fossa, the level of calcification in the condylar head, and the temporomandibular joint spaces. According to the Diagnostic Criteria for Temporomandibular Disorders (DC/TMD) guidelines, the diagnosis of degenerative joint disease can be confirmed if computed tomography (CT) imaging reveals at least one of the following features: subchondral cysts, erosions, generalized sclerosis, or osteophytes [4,20,29]. This 3D imaging method inherently removes superimposition and distortion, providing high-spatial-resolution images. Other benefits of CBCT include shorter scan times and lower radiation doses compared to other methods, given that the appropriate imaging parameters are used. CBCT is a dependable method for analyzing the bony structures of the TMJ in coronal, axial, and sagittal views. In CBCT, images can be reconstructed within the image volume for easier visualization of the TMJ [19,20,25,30].

TMJ evaluation in the US includes assessing abnormalities in disk positioning, joint effusion, and bone pathologies [31]. One of the primary benefits of ultrasound is its non-invasive nature; it uses high-frequency sound waves to create images of internal structures, making it a safer and more comfortable option for patients. The ultrasound image is interpreted in real time as it is obtained, which places a considerable demand on the radiologist’s expertise and experience [32,33]. This immediate interpretation is crucial because it allows for dynamic assessment of the patient’s condition, providing instant feedback that can be essential for guiding further diagnostic or therapeutic decisions [16,34].

Magnetic resonance imaging is widely regarded as the preferred imaging technique for the temporomandibular joint due to its exceptional ability to provide detailed and comprehensive views of both the bony and soft tissue structures within the joint. MRI’s superior contrast resolution allows for precise assessment of the TMJ’s intricate components, making it invaluable in diagnosing and managing a range of TMDs [2,32,35]. Specifically, MRI is instrumental in evaluating the bony structures of the TMJ, including the mandibular condyle and the temporal bone, as well as the surrounding soft tissues. It excels in visualizing the masticatory muscles such as the masseter, temporalis, and lateral pterygoid muscles, which play a crucial role in jaw movement and function. Additionally, MRI provides clear images of the ligaments associated with the TMJ, such as the lateral and sphenomandibular ligaments, which are essential for joint stability and function. One of the key advantages of MRI is its ability to depict the cartilaginous disc of the TMJ with high accuracy. This disc, which acts as a cushion between the mandibular condyle and the temporal bone, is critical for smooth and pain-free jaw movement. MRI can reveal disc displacement, deformation, and degeneration, which are often implicated in TMD [16,19].

We focused on evaluating the temporomandibular joint using ultrasonography, cone-beat computed tomography, and magnetic resonance imaging.

The latest meta-analysis on bruxism conducted in 2024 by Zieliński et al. [36] observed a prevalence of bruxism at 21%, with a higher prevalence of 23% specifically for bruxism occurring while awake. In our study, a significant association was observed between teeth grinding (bruxism) and alterations in bone structures, as evidenced by CBCT scans (*p* = 0.027). Bone structures may be less susceptible to the mechanical stresses imposed by bruxism, or alternatively, it may be that bruxism contributes to the preservation of bone structure through adaptive remodeling processes. It aligns with previous research indicating that bruxism is a significant contributing factor to joint wear and morphological changes. The high prevalence of teeth grinding in patients with compromised bone integrity may reflect the role of bruxism in exacerbating structural degradation through repetitive mechanical loading and microtrauma [36]. Furthermore, the adaptive response of the temporomandibular joint to bruxism-induced stress might involve compensatory changes in bone density and structure which could be visualized as alterations in radiographic images [34]. Yamada et al. [37] identified a link between condylar alterations and “parafunctional” habits. They concluded that increased intensity and frequency of muscular activity elevate the risk of condylar changes and TMJ degeneration.

Our study revealed significant associations between structural abnormalities and limited mandibular opening, as identified through ultrasonography and magnetic resonance imaging. The MRI findings further corroborated these associations, although with some differences that underscore the impact of bone structure integrity on mandibular function. Additionally, the presence of right disc dislocation was associated with limited mandibular opening. Disc dislocation significantly contributes to mechanical and functional disturbances, leading to restricted mandibular movement. The observed associations highlight the critical role of structural abnormalities in the etiology of limited mandibular opening [21,34,38]. Erosions in the mandibular head and abnormal bone structures likely disrupt the smooth articulation of the temporomandibular joint, leading to mechanical impedance and pain that restrict jaw movement. The presence of disc dislocation further exacerbates these issues by destabilizing the joint, thereby increasing the likelihood of limited mandibular opening. Our results align with previous studies that have demonstrated the impact of TMJ structural abnormalities on mandibular function. Research has shown that degenerative changes in the TMJ such as erosions and bone abnormalities are often associated with pain and limited jaw movement. Disc dislocations have also been identified as a common cause of TMJ dysfunction, leading to symptoms such as clicking, pain, and restricted opening [1,18,21,34].

Using cone-beam computed tomography, it was found that patients with a narrowed left joint space demonstrated a significantly higher prevalence of right side muscle tension. This finding indicates that reduced joint space may contribute to increased muscle strain, likely due to altered joint function and increased load on the surrounding musculature. Imbalance within the joint can lead to compensatory muscle activity and subsequent tension. Our observations agree with those of Lauriti et al. [39]. In their research, they demonstrated a link between increased tension in the masseter and temporal muscles in individuals with TMD. The study revealed statistically significant differences in the electromyographic activity of these muscles in the resting position among adolescents with moderate to severe TMD compared to those with mild TMD and those without the condition [39,40]. The presence of erosions in the right mandibular head also showed a significant association with right side masticatory muscle tension. This association underscores the potential role of joint degenerative changes in the etiology of muscle tension, likely due to the disruption of normal joint architecture and the resultant abnormal loading patterns on the masticatory muscles. Lekroengsin et al. [41] demonstrated that changes in the mandibular condyle are also influenced by variations in the volume of the medial pterygoid muscle. Ultrasonography observations corroborated these findings. This consistency across imaging modalities reinforces the validity of our results and highlights the utility of both CBCT and US in the assessment of TMD. These findings are consistent with the existing literature, which suggests that joint pathology can lead to altered joint mechanics and increased muscle strain [34].

Acoustic changes were reflected in radiological alterations across all three imaging modalities. Erosions detected by CBCT and MRI were strongly associated with acoustic changes. This relationship may be explained by a biomechanical or compensatory mechanism where structural damage on one side of the jaw alters movement dynamics on the opposite side, leading to audible joint sounds. Asymmetrical biomechanical stresses can prompt compensatory adjustments that manifest as these acoustic symptoms [42]. Findings from ultrasonography further corroborate the connection between TMJ abnormalities and acoustic symptoms. The reduction in joint pain symptoms, despite increasing damage and sounds from the joint, can be attributed to the joint’s gradual adaptation to pathological changes. Despite significant damage, the condition stabilizes, aligning with the understanding that TMD is a chronic condition requiring ongoing monitoring of bone changes and joint adaptation [43]. In the US, a normal outline of the right mandibular head was associated with a higher incidence of acoustic changes, suggesting that soft tissue conditions or functional dynamics play a crucial role in the development of these symptoms. The lower incidence of acoustic symptoms in the presence of degenerative changes may be due to decreased joint mobility or altered mechanics, which reduce dynamic interactions within the joint and thus produce less noise [32,34,38,44,45,46]. The absence of joint clicking and crepitation does not necessarily indicate a normal joint, nor does the presence of joint sounds automatically signify disease [43]. MRI analysis revealed significant associations between the condition of the right articular disc and the prevalence of acoustic changes on the left side. These results suggest that the integrity and uniformity of the articular disc are crucial in the pathophysiology of temporomandibular joint disorders and their associated symptoms [32]. Previous studies have highlighted the role of disc displacement and heterogeneity in contributing to TMDs and related symptoms. Manfredini et al. [34] demonstrated that disc displacement, especially when accompanied by structural abnormalities, significantly contributes to the development of TMD symptoms, including joint sounds and pain. Our findings align with these observations, indicating that a homogeneous disc structure is essential for minimizing the risk of acoustic changes. A homogeneous disc may provide more consistent cushioning and distribution of mechanical forces, thereby reducing abnormal joint sounds and enhancing overall joint function. Conversely, a heterogeneous disc may lead to uneven force distribution and increased joint friction, contributing to the development of acoustic symptoms [44,47].

The relationship between radiologically assessed bone structures and the occurrence of pain in temporomandibular joint disorders is multifaceted and significant. The presence of radiologically normal bone structures does not necessarily preclude the occurrence of pain, suggesting that other factors, such as soft tissue conditions or functional dynamics, might play a significant role [38,48]. Condylar flattening showed the strongest correlation with overall pain complaints [48]. In our study, erosions in the bone structures were associated with a significantly lower incidence of reported pain. That could imply that erosions may lead to adaptive changes that reduce pain perception or that pain may prompt individuals to alter their jaw movements, thereby mitigating further discomfort [38,48]. Narrowed left joint space was linked with a higher incidence of pain. This further underscores the importance of joint space symmetry and adequate spacing in preventing pain. This could reflect the compensatory or biomechanical effects of right side abnormalities influencing the contralateral side’s function and pain perception [13,34,38]. MRI findings corroborated the significance of disc morphology, showing that heterogeneous left articular discs were more often associated with pain (70% vs. 30%), mirroring the US results. This supports the notion that disc composition and integrity are critical factors in the development of pain symptoms. The heterogeneous discs likely indicate degenerative changes or abnormal biomechanics, which can exacerbate pain. Our findings align with the broader literature on TMD, which emphasizes the complex interplay between structural, functional, and possibly psychosocial factors in the manifestation of pain [32,34,38,46,49]. Manfredini et al. [34] highlight that while structural abnormalities are important, the subjective experience of pain in TMD often correlates more strongly with functional disruptions and adaptive changes within the joint.

Similarly to pain, palpation discomfort was also reflected in all three imaging modalities. The presence of erosions in the right mandibular head, as detected by CBCT, is significantly associated with increased palpation discomfort, consistent with previous studies indicating that structural changes in the mandibular head, such as erosions, are frequently linked to pain and discomfort due to underlying degenerative or inflammatory processes [33]. Interestingly, a normal outline of the left mandibular head, as revealed by US, correlates with a higher incidence of palpation discomfort on the right side. This might suggest compensatory mechanisms or asymmetrical load distribution between the joints, indicating that even a normal-appearing mandibular head on one side may be associated with dysfunction or compensatory stress on the contralateral side [33]. MRI findings demonstrate a significant association between erosions in the right bone structures and palpation discomfort. This strong correlation underscores the sensitivity of MRI in detecting subtle structural changes that might not be evident in other imaging modalities, thereby highlighting its importance in diagnosing TMD-related discomfort [50,51]. The presence of disc dislocation on the right side also shows a significant correlation with increased palpation discomfort. These findings are consistent with existing literature that associates disc dislocation with mechanical interference, inflammation, and subsequent pain and discomfort in TMD patients [35].

One limitation of our study is the sample size and the imbalance in the number of participants across different groups. The unequal group sizes may affect the generalizability of the findings and potentially introduce bias. Another limitation is that not all patients underwent all the imaging modalities.

Our study distinguishes itself from the existing literature by providing a comprehensive evaluation of multiple aspects using three different imaging modalities. We did not find any articles that compare clinical symptoms across CBCT, MRI, and US.

## 5. Conclusions

In our study, we observed a range of clinical symptoms that could be compared with radiological images. CBCT revealed statistically significant changes in patients with bruxism, while MRI and ultrasound imaging showed changes in patients with limited jaw opening. Muscle tension was evident in both US and CBCT images. Acoustic changes, such as temporomandibular joint pain or palpation, were visible in all three imaging methods. These findings highlight the importance of detailed imaging and structural assessment for the effective diagnosis and management of temporomandibular joint disorders. Depending on the patient’s reported symptoms, various alterations may be observed in radiographic images. Imaging studies serve as valuable supplementary diagnostic tools for TMD and should be conducted based on both subjective and objective clinical assessments.

One of the primary constraints is the small sample size, which may reduce the statistical power of our findings and limit the generalizability of the results to a broader population. The uneven distribution of participants among the different groups could potentially skew the data, leading to less reliable comparisons between the imaging methods. Another limitation is that not all patients underwent every imaging modality.

Given the broad range of TMD symptoms and the various diagnostic options available, future studies should include a larger and more diverse patient population. This would ensure a more balanced distribution of groups and provide a more comprehensive understanding of the disorder. In the future, clinicians would benefit from having clearer guidelines and standardized protocols for diagnosing TMD, which would facilitate decision making regarding the appropriate diagnostic approaches.

## Figures and Tables

**Table 1:** The protocol used during MRI.

SEQUENCES	TR	TE	SLICE (mm)	FOV	MATRIX
T2 TSE AXIAL	3000–4000	100–120	3	210–230	320 × 320
PD TSE SPAIR CORONAL	2500–3500	15–30	2	100	256 × 256
PD TSE SPAIR SAGITTAL (close mouth)	2500–3500	15–30	2	100	256 × 256
STIR SAGITTAL (close mouth)	3000–4000	90–100	2	100	256 × 224
PD SAGITTAL (open mouth)	2500–3500	15–30	2	100	256 × 256

**Table 2:** Demographic profile of patients in the study sample.

Characteristic	*n*	Distribution ^a^
Age, years	63	39.00 (28.50, 54.50) ^b^
Sex	63	
female		51 (80.95%)
male		12 (19.05%)
Disorder duration	63	
up to 6 months		23 (36.51%)
over 6 months		40 (63.49%)

^a^ n (%). ^b^ Mdn (Q1, Q3). Note: *n*—sample size; Mdn—median; Q1—the first quartile (25%); Q3—the third quartile (75%).

**Table 3:** The association between the radiological findings and clinical manifestations of limited mandibular opening among patients with TMD.

		Limited Mandibular Opening			
Radiological Findings	*n*	Yes n_2_ = 14 ^a^	No n_1_ = 49 ^a^	*p* ^b^	ϕ
US: right mandibular head—erosions present	52	4 (33.33%)	1 (2.50%)	0.008	0.44
MRI: right bone structures—normal	46	1 (11.11%)	23 (62.16%)	0.009	0.41
MRI: right bone structures—abnormal	46	8 (88.89%)	14 (37.84%)	0.009 ^c^	0.41
MRI: conclusion—right disc dislocation	46	3 (33.33%)	2 (5.41%)	0.044	0.36

^a^ n (%). ^b^ Fisher’s exact test. ^c^ Pearson’s Chi-squared test.

**Table 4:** The association between the radiological findings and clinical manifestations of right side masticatory muscle tension among patients with TMD.

		Right Side Masticatory Muscle Tension			
Radiological Findings	*n*	Yes n_2_ = 12 ^a^	No n_1_ = 51 ^a^	*p* ^b^	ϕ
CBCT: left joint space—normal	48	2 (22.22%)	26 (66.67)	0.024	0.35
CBCT: left joint space—narrowed	48	6 (66.67%)	9 (23.08%)	0.018 ^c^	0.37
CBCT: left joint space—asymmetrical	48	6 (66.67%)	6 (15.38%)	0.004 ^c^	0.46
CBCT: right mandibular head—erosions present	48	5 (55.56%)	6 (15.38%)	0.020 ^c^	0.37
CBCT: left bone structures—normal	48	5 (55.56%)	35 (89.74%)	0.031 ^c^	0.36
US: right bone structures—erosions present	52	3 (37.50%)	3 (6.82%)	0.040	0.35

^a^ n (%). ^b^ Fisher’s exact test. ^c^ Pearson’s Chi-squared test.

**Table 5:** The association between the radiological findings and clinical manifestations of acoustic changes (right side) among patients with TMD.

		Acoustic Changes (Right Side)			
Radiological Findings	*n*	Yes n_2_ = 29 ^a^	No n_1_ = 34 ^a^	*p* ^b^	ϕ
CBCT: left mandibular head—erosions present	48	8 (34.78%)	0 (0.00%)	0.001 ^c^	0.47
US: right mandibular head—normal outline	52	20 (83.33%)	16 (57.14%)	0.041	0.28
US: right mandibular head—abnormal outline	52	4 (16.67%)	12 (42.86%)	0.041 ^c^	0.28
US: conclusion—degenerative change in the right joint	52	8 (33.33%)	17 (60.71%)	0.049	0.27

^a^ n (%). ^b^ Fisher’s exact test. ^c^ Pearson’s Chi-squared test.

**Table 6:** The association between the radiological findings and clinical manifestations of acoustic changes (left side) among patients with TMD.

		Acoustic Changes (Left Side)			
Radiological Findings	*n*	Yes n_2_ = 28 ^a^	No n_1_ = 35 ^a^	*p* ^b^	ϕ
US: right articular disc—homogeneous	52	15 (65.22%)	10 (34.48%)	0.028	0.31
US: right articular disc—heterogeneous	52	8 (34.78%)	19 (65.52%)	0.028	0.31
MRI: right articular disc—homogeneous	46	18 (90.00%)	12 (46.15%)	0.002	0.46
MRI: right articular disc—heterogeneous	46	2 (10.00%)	14 (53.85%)	0.002 ^c^	0.46

^a^ n (%). ^b^ Fisher’s exact test. ^c^ Pearson’s Chi-squared test.

**Table 7:** The association between the radiological findings and clinical manifestations of pain (left joint) among patients with TMD.

		Pain (Left Joint)			
Radiological Findings	n	Yes n_2_ = 33 ^a^	No n_1_ = 30 ^a^	*p* ^b^	ϕ
CBCT: right joint space—asymmetrical	48	8 (40.00%)	4 (14.29%)	0.043 ^c^	0.29
CBCT: left joint space—normal	48	8 (40.00%)	20 (71.43%)	0.029	0.31
CBCT: left joint space—narrowed	48	10 (50.00%)	5 (17.86%)	0.018	0.34
CBCT: left joint space—asymmetrical	48	8 (40.00%)	4 (14.29%)	0.043 ^c^	0.29
CBCT: right articular tubercle—normal outline	48	20 (100.00%)	22 (78.57%)	0.034	0.32
CBCT: right articular tubercle—abnormal outline	48	0 (0.00%)	6 (21.43%)	0.034 ^c^	0.32
US: right bone structures—normal	52	13 (48.15%)	22 (88.00%)	0.002	0.42
US: left articular disc—homogeneous	52	8 (29.63%)	15 (60.00%)	0.028	0.31
US: left articular disc—heterogeneous	52	19 (70.37%)	10 (40.00%)	0.028	0.31
US: right mandibular head—normal outline	52	14 (51.85%)	22 (88.00%)	0.005	0.39
US: right mandibular head—abnormal outline	52	13 (48.15%)	3 (12.00%)	0.005 ^c^	0.39
MRI: left articular disc—homogeneous	46	6 (30.00%)	17 (65.38%)	0.017	0.35
MRI: left articular disc—heterogeneous	46	14 (70.00%)	9 (34.62%)	0.017	0.35

^a^ n (%). ^b^ Fisher’s exact test. ^c^ Pearson’s Chi-squared test.

**Table 8:** The association between the radiological findings and clinical manifestations of palpation discomfort (right side) among patients with TMD.

		Palpation Discomfort (Right Side)			
Radiological Findings	n	Yes n_2_ = 37 ^a^	No n_1_ = 26 ^a^	*p* ^b^	ϕ
CBCT: right mandibular head—erosions present	48	4 (12.90%)	7 (41.18%)	0.036	0.32
US: left mandibular head—normal outline	52	16 (50.00%)	16 (80.00%)	0.031 ^c^	0.30
MRI: right bone structures—erosions present	46	0 (0.00%)	3 (20.00%)	0.030	0.38
MRI: conclusion—right disc dislocation	46	1 (3.23%)	4 (26.67%)	0.033	0.35

^a^ n (%). ^b^ Fisher’s exact test ^c^ Pearson’s Chi-squared test.

**Table 9:** The association between the radiological findings and clinical manifestations of palpation discomfort (left side) among patients with TMD.

		Palpation Discomfort (Left Side)			
Radiological Findings	n	Yes n_2_ = 22 ^a^	No n_1_ = 41 ^a^	*p* ^b^	ϕ
CBCT: right joint space—asymmetrical	48	7 (50.00%)	5 (14.71%)	0.024	0.37
CBCT: left joint space—normal	48	5 (35.71%)	23 (67.65%)	0.041	0.29
CBCT: left joint space—asymmetrical	48	7 (50.00%)	5 (14.71%)	0.024	0.37
US: right bone structures—normal	52	8 (44.44%)	27 (79.41%)	0.011	0.36
US: left articular disc—homogeneous	52	4 (22.22%)	19 (55.88%)	0.020 ^c^	0.32
US: left articular disc—heterogeneous	52	14 (77.78%)	15 (44.12%)	0.020	0.32
US: right mandibular head—normal outline	52	9 (50.00%)	27 (79.41%)	0.029	0.30
US: right mandibular head—abnormal outline	52	9 (50.00%)	7 (20.59%)	0.029	0.30
US: right mandibular head—erosions present	52	4 (22.22%)	1 (2.94%)	0.043 ^c^	0.31
US: conclusion—right disc edema	52	4 (22.22%)	1 (2.94%)	0.043 ^c^	0.31
MRI: left bone structures—erosions present	46	3 (25.00%)	0 (0.00%)	0.014 ^c^	0.45

^a^ n (%). ^b^ Fisher’s exact test ^c^ Pearson’s Chi-squared test.

## Data Availability

The data on which this study is based will be made available upon request.
